# Spatial-temporal variability and influence factors of Cd in soils of Guangxi, China

**DOI:** 10.1371/journal.pone.0279980

**Published:** 2023-01-10

**Authors:** Mi Tian, Xueqiu Wang, Futian Liu, Qinghai Hu, Yu Qiao, Qiang Wang

**Affiliations:** 1 Key Laboratory of Geochemical Exploration, Institute of Geophysical and Geochemical Exploration, CAGS, Langfang, Hebei, China; 2 UNESCO International Center on Global-scale Geochemistry, Langfang, Hebei, China; 3 Lanzhou University & Key Laboratory of Strategic Mineral Resources of the Upper Yellow River, School of Earth Sciences, Ministry of Natural Resources, Lanzhou, Gansu, China; Tsinghua University, CHINA

## Abstract

In this study, the regional spatial-temporal variability of cadmium (Cd) in the topsoil of Guangxi, China from 2010 to 2016 was studied from data obtained from the China Geochemical Baseline Project (CGB Ⅰ and CGB Ⅱ). The driving forces of natural and anthropogenic variables were quantitatively analyzed using a geographically and temporally weighted regression model. The results showed that 1) soil Cd was highly enriched in 2010 and in soils of Hechi city in northwest Guangxi, a non-ferrous metal mining and metallurgy area, ~17% of the samples exceeded the soil contamination risk limit. In contrast, in 2016, the topsoil Cd content decreased significantly, with 7% of sites exceeding the soil risk limit. 2) Multiple factors jointly influenced the regional spatial variability of Cd. pH and organic carbon were found to be the main factors influencing Cd content and were strongly spatially correlated with Cd. Anthropogenic activities, including mining and industrial emissions, resulted in significant Cd enrichment in local areas, whereas agricultural and domestic pollutants were relatively weakly correlated with Cd. The weathering products of carbonates were significantly enriched in Cd; thus, the geological background played a significant role in the spatial variability of Cd. Soil-forming factors, including temperature, precipitation, and elevation influenced the spatial distribution of Cd, especially in the Cd background area. 3) Anthropogenic activities were the key factors influencing temporal changes in Cd. Mining caused significant enrichment of Cd in CGB Ⅰ, while industrial emissions were the primary factor for Cd enrichment in CGB Ⅱ. In addition, natural factors also played an important role; the increased Normalized Difference Vegetation Index suggested reduced desertification and reduction of soil erosion in the watershed and in pollutants transported from upstream.

## 1 Introduction

Cadmium (Cd) is an extremely dispersed heavy metal with high toxicity in humans, causing kidney failure, hematuria, and bone disease if excessively ingested [1,2]. Cd content in soils is often influenced by parent materials, geochemical processes such as leaching and transfer through surface water runoff, and anthropogenic inputs [3–7]. Soil-forming parent materials provide the initial sources of Cd in soils, which may lead to enrichment or loss of elements, and anthropogenic inputs include mining, industrial emissions, and agricultural activities [8,9]. Therefore, multiple factors may cause spatial heterogeneity of Cd in soil. Meanwhile, Cd concentrations in the same area or at the same sampling site may vary temporally due to anthropogenic or natural environmental changes [10–12]. Studying the spatial-temporal variability of Cd and its driving factors at a regional scale provides insight into soil Cd content, quantifies the influencing factors to enable prediction of changes in soil Cd, and informs the evaluation and regulation of soil Cd pollution.

Previous studies on the spatial-temporal variability of soil heavy metals are based on geostatistical methods comparing spatial interpolation maps over different periods, including inverse distance weighting, kriging, and stochastic simulation [13–15]. However, these methods do not consider the influence of environmental and anthropogenic driving variables on heavy metal content. Soil source apportionment methods usually include isotope tracing [16], principal component analysis [17], positive matrix factorization (PMF) [18], chemical mass balance [19], self-organizing maps (SOM) [20], UNMIX receptor models [21], and machine learning, including support vector machines [22], artificial neural networks [23], and random forests [24]. These methods are widely used in static status analysis. However, the sources of heavy metals may vary spatially and temporally, which is not captured by traditional methods. [25] proposed a geographically weighted regression model (GWR) that considered the non-stationarity nature of the data and allowed for quantitative analysis of relationships between two or more variables through regression. This approach allows for a local rather than a global fit to the data and exploration of the spatial relationships between the explanatory and dependent variables. [26] proposed a geographically and temporally weighted regression (GTWR) model capturing the parameter variation in both temporal and spatial dimensions. GTWR has been successfully applied to the study of carbon emissions, aerosols, energy efficiency, and housing prices [27,28]. However, until now, GTWR has not been applied to the spatial-temporal variability of heavy metals.

The Global Geochemical Baselines (GGB) project aims to provide a long-term and effective quantitative scale for resource and environmental assessment. The GGB project divides the world into several grids of a certain size and collects representative samples to reflect the overall element levels of the grid [29,30]. As a part of the GGB project, the far-reaching China Geochemical Baselines (CGB) resources and environmental projects were completed in 2008–2012 (CGB I) and 2015–2019 (CGB II), using 81 geochemical parameters [29,31,32]. Guangxi Province in China is rich in mineral resources particularly non-ferrous metals. Lead-zinc (Pb-Zn) ores, as the main occurrence medium of Cd, are abundant in Guangxi province. Studies show that pollutants from mining have led to serious soil Cd contamination in many areas of Guangxi [33–35]. Guangxi is a typical karst region with widely distributed carbonates. The weathering products of these carbonates are relatively enriched in Cd due to the unique geochemical properties and the high temperature and rainfall climate in Guangxi. Thus, soils in most areas of Guangxi usually show high background values of Cd [36]. Furthermore, industrial emissions, agricultural pollutants, and domestic pollutants may lead to Cd enrichment in the topsoil. As a result, multiple complex factors control the distribution of soil Cd in this area. In this study, a spatial-temporal model was constructed using GTWR to investigate the spatial-temporal variability (2010–2016) and driving factors of Cd in soils of Guangxi at a regional scale based on the data from CGB Ⅰ and CGB Ⅱ. The study aimed to understand the sources and changing trends of Cd and to provide a geochemical basis for the prevention and control of Cd pollution in Guangxi.

## 2 Materials and methods

### 2.1 Study area

Guangxi province, located in south China (104°26’E–112°04’E, 20°54’N–26°24’N), has a total area of ~23.67 × 104 km2 and has a subtropical monsoon humid climate. The terrain inclines from the northwest to the southeast. Karst landforms are widely distributed in central, western, and northwestern Guangxi, with an area of approximately 9.58 × 104 km^2^, accounting for 40.9% of the total area.

### 2.2 Materials and sampling

The CGB Ⅰ, as part of the Global Geochemical Baselines project [37], was carried out from 2008 to 2012 [37]. Alluvial soil samples were collected according to the global reference grid cell network which covers an area of 9.6 million km^2^ in mainland China. The CGB II was conducted from 2015 to 2019.

The temporal change of Cd concentrations may increase or decrease, a major issue is whether these changes are detectable by soil monitoring taking. Sampling materials and monitoring sites must indicate that contaminants can build quickly enough to be revisited on subsequent occasions [38]. Transported samples collected from the outlets of large drainage catchments represent the natural background and anthropogenic Cd content. Contamination of alluvial soils can occur relatively quickly. Diffuse sources, such as natural weathering, mining, industries, residents, pesticides, and fertilizers, contribute to pollution. Pollutants are transported by rainfall runoff into watercourses and deposited in low-reach plains, overbanks, or fluvial terraces. The sampling media, sampling methods, analytical laboratories, analytical methods, and quality control were identical for CGB Ⅰ and CGB II, making the data comparable. In this study, the CGB Ⅰ samples at the same site was selected according to the CGB II sampling sites, or the element contents of the CGB Ⅰ samples in the same watershed of CGB II sampling site were averaged to represent the element contents of the watershed.

### 2.3 Sample preparation and laboratory analysis

The samples were prepared and subjected to chemical analysis in a single laboratory. The samples were air-dried and homogenized, and each raw sample was split into two sub-samples, one by passing through 10 mesh (<2 mm) for laboratory analysis, and the other for storage and future investigation. The sieved sample was ground through a 200 mesh (<74 μm) in an agate mill for laboratory analysis.

An aliquot (0.25 g was weighed and placed into a test tube, and 10 mL HF, 5 mL of HNO_3_, 2 mL of HClO_4_ were added to digest the samples. The test tube was heated in a boiling water bath until dry. After cooling, 8 ml of 1:1 aqua regia [aqua regia (1HNO_3_ + 3 HCl): pure water = 1:1 vol.] was then added to decompose the residue. The solution was diluted with 2% HNO_3_. The solution was then analyzed using ICP-MS [39]. The detection limit was 0.02 mg/kg. The accuracy of the method was assessed 34 times by analyzing the soil reference materials (GSS-1, GSS-2, GSS-17, GSS-19, GSS-25, GSS-26, GSS-27) [40], and the △lgC was less than 0.10 (△lgC = |lgC_i_—lgC_s_|, C_i_ is the average of measured values; C_s_ is the standard reference value).

### 2.4 Factor selection and data processing

Parameter selection was based on the relevance of the factors to the spatial-temporal variability of Cd, availability, and quantifiability. In this study, 11 variables were selected: pH, organic carbon, geological background (represented by Zr content), temperature, precipitation, elevation, normalized difference vegetation index (NDVI), industrial emissions, agricultural pollutant emissions, domestic pollutant emissions, and deposit density (representing the intensity of mining activities).

**Fig 1:**
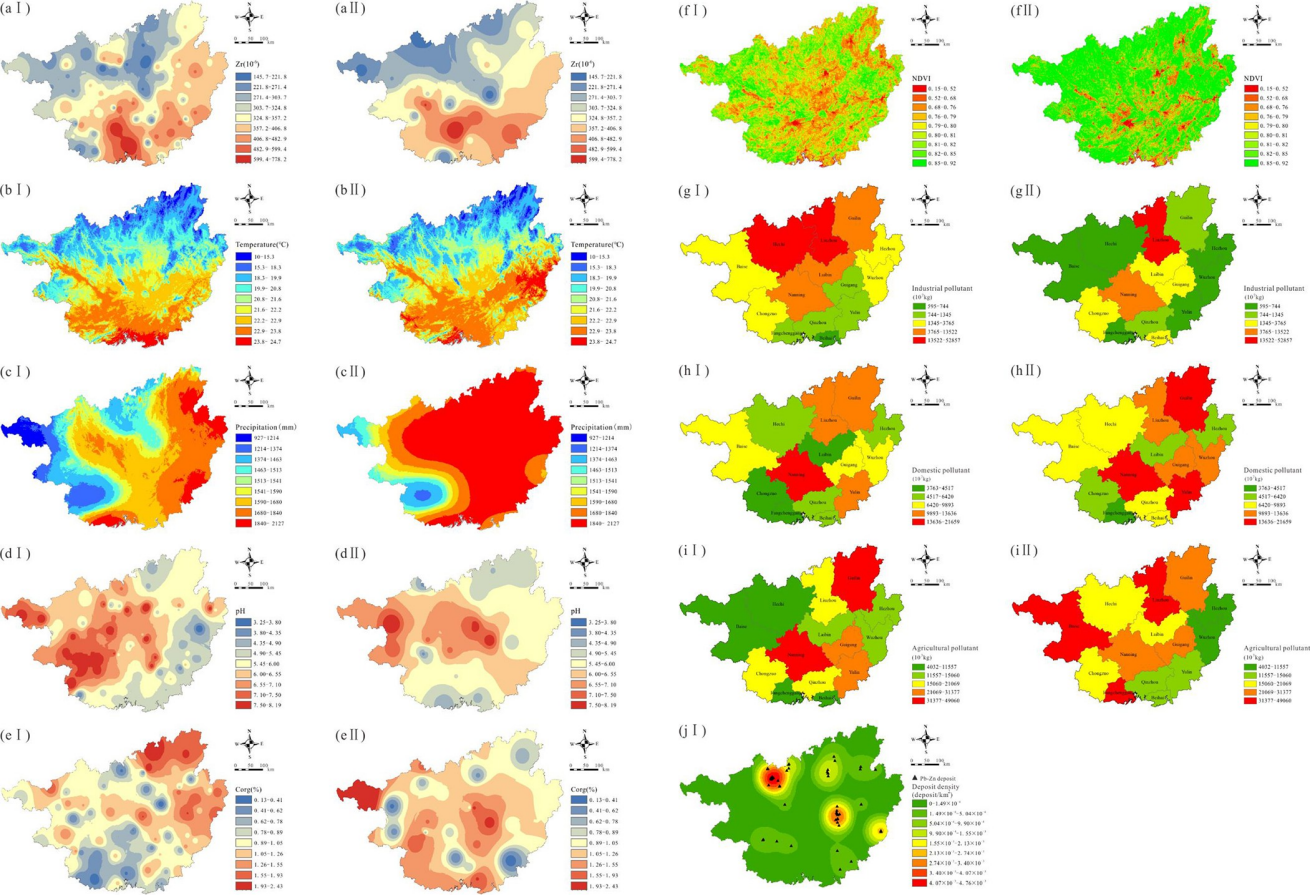
Spatial-temporal distribution of driving factors (a: Zr; b: Temperature; c: Precipitation; d: pH; e: Corg (organic carbon); f: NDVI; g: Industrial pollutants; h: Domestic pollutants; i: Agricultural pollutants; j: Deposit density; Ⅰ for CGB Ⅰ; Ⅱ for CGB Ⅱ).

Zr, which is geochemically stable and uneasily migrates during weathering [41], was chosen as a proxy for the geological background. Organic carbon, pH, and Zr content were analyzed in the laboratory. The digital elevation model data set was provided by Geospatial Data Cloud site, Computer Network Information Center, Chinese Academy of Sciences (http://www.gscloud.cn). Raster data for the annual average NDVI, temperature, and precipitation for 2010 (CGB I) and 2016 (CGB II) were collected from the Resource Environmental Science and Data Center, Institute of Geographical Sciences and Natural Resources Research, Chinese Academy of Sciences (https://www.resdc.cn/data.aspx?DATAID=123). The coordinates of the deposits in Guangxi were obtained from the website of the Guangxi Hechi Ecological Environment Bureau (http://sthjj.hechi.gov.cn/); locations of the mining deposits remained fairly unchanged from 2010 to 2016. The spatial analysis module of ArcGIS 10.4 was used to calculate the kernel density, and then the deposit density for each sampling site was obtained by extracting the value to point data. Industrial wastewater discharge, domestic wastewater discharge, and agricultural pollutant discharge (sum of chemical oxygen demand discharge and ammonia nitrogen discharge) were obtained from the 2010 and 2016 statistical yearbooks of the Guangxi Province. The spatial-temporal distribution of the 11 variables is shown in Fig 1.

Box–Cox transformation was applied to both the dependent and independent variables to transform the data to an approximate normal distribution to achieve the best result. The independent variables (influencing factors) were standardized to allow for comparison of coefficients of each variable.

### 2.5 Principles of GTWR

**1) GWR model.** The formula for the GWR model is [42]:

$$Y_{i}=\beta_{0}(\mu_{i},\nu_{i})+\sum_{k}\beta_{k}(\mu_{i},\nu_{i})X_{i}{}_{k}+\varepsilon_{i}$$ (1)

where *Y*_*i*_ is the independent variable at sample site *i*, *X*_*ik*_ is the *k*th dependent variable at sample site *i*, *μ*_*i*_ is the longitude coordinate of sample site *i*, *ν*_*i*_ is the latitude coordinate of sample site *i*, (*μ*_*i*_, *ν*_*i*_) is the spatial latitude and longitude coordinate of sample site *i*, *β*_0_(*μ*_*i*_, *ν*_*i*_) is the constant term at sample site *i*, *β*_*k*_(*μ*_*i*_, *ν*_*i*_) is the regression coefficient of the *k*th dependent variable at sample site *i*, and *ε*_*i*_ is the random error.

The regression coefficients of GWR are estimated separately for each spatial site, whereas the regression coefficients of the ordinary linear regression model are estimated based on the entire study area [43].

**2) GTWR model.** The GTWR embeds the dimension of temporal data into regression parameters to measure both spatial and temporal variations. The structure of the GTWR model is as follows:

$$Y_{i}=\beta_{0}(\mu_{i},\nu_{i},t_{i})+\sum_{k}\beta_{k}(\mu_{i},\nu_{i},t_{i})X_{i}{}_{k}+\varepsilon_{i}$$ (2)

where *Y*_*i*_ is the independent variable of the *i*th sample point; *X*_*ik*_ is the *k*th dependent variable of the *i*th sample point; *μ*_*i*_ is the longitude coordinate of the *i*th sample point; *ν*_*i*_ is the latitude coordinate of the *i*th sample point; *t*_*i*_ is the spatial latitude and longitude coordinates of the *i*th sample point; (*μ*_*i*_, *ν*_*i*_, *t*_*i*_) is the spatial and temporal dimension coordinates of the *i*th sample point; *β*_0_(*μ*_*i*_, *ν*_*i*_, *t*_*i*_) is the constant term of the *i*th sample point; *β*_*k*_(*μ*_*i*_, *ν*_*i*_, *t*_*i*_) is the regression coefficient of the *k*th independent variable at the *i*th sample point, which reflects the spatial-temporal differentiation of the influence of different variables. The positive and negative signs of the coefficients indicate the positive and negative correlations between the variables, respectively, and the magnitude indicates the strength of the correlation; *ε*_*i*_ is the random error.

Similar to GWR, the regression coefficients for GTWR are estimated based on locally weighted least squares, with the parameters estimated as follows:

$$\hat{\beta }(\mu_{i},\nu_{i},t_{i})={\left[X^{T}W(\mu_{i},\nu_{i},t_{i})X\right]}^{-1}X^{T}W(\mu_{i},\nu_{i},t_{i})Y$$ (3)

where the spatial-temporal weight matrix *W*(*μ*_*i*_, *ν*_*i*_, *t*_*i*_) is a diagonal matrix, $W(\mu_{i},\nu_{i},t_{i})=diag(W_{i1},W_{i2},\cdots ,W_{ij},\cdots ,W_{in})$ and *W*_*ij*_(1≤*j*≤*n*) is the spatial-temporal distance decay function calculated as follows:

$$W_{i}{}_{j}=\exp[-\frac{{\left(d_{ij}^{ST}\right)}^{2}}{h^{2}}]$$ (4)

where $d_{ij}^{ST}$ is the spatial-temporal distance, calculated as follows:

$$d_{ij}^{ST}=\sqrt{\lambda [{\left(\mu_{i}-\mu_{j}\right)}^{2}-{\left(v_{i}-v_{j}\right)}^{2}]+\mu {\left(t_{i}-t_{j}\right)}^{2}}$$ (5)

where *λ* and *μ* are scaling factors that balance the temporal and spatial distances, *h* is the spatial-temporal bandwidth, including the fixed and adaptive bandwidths. Adaptive bandwidth is widely used because it can be adjusted adaptively according to the distributions of sample sites around the regression point, taking a larger bandwidth when there are fewer sample points nearby and using a smaller bandwidth when there are more sample points nearby.

The optimal bandwidth was selected based on the minimum variance verification (CV) value, which is the sum of the squared errors between the actual and predicted values.


$$CV(h)=\sum_{i}{\left(y_{i}-{\hat{y}}_{i}(h)\right)}^{2}$$ (6)

AICc is a common metric used in final model decisions, and the model with the lowest AICc value was selected. In this research, model parameters were estimated using the GTWR in ArcGIS 10.4 [26], with automatic optimal settings for bandwidth and spatial-temporal distance scaling factors.

## 3 Results

### 3.1 Descriptive statistical analysis of Cd content

Descriptive statistical analyses of the Cd content in CGB Ⅰ and CGB Ⅱ are shown in Table 1. The median value of Cd for CGB Ⅰ was 295.45 μg/g, and the median value of Cd for CGB Ⅱ was 236.24 μg/g, slightly lower than that of CGB Ⅰ. Both values were higher than the median soil Cd values for China, the continental crust, and Europe, and slightly lower than that of European floodplain sediments. The average values of Cd for CGB I and CGB II were 1131.55 μg/g and 392.68 μg/g, respectively. Both values were higher than the average Cd soil content of China and Europe, while the CGB Ⅱ Cd average value was lower than the European floodplain sediment Cd average value. The maximum value of Cd in CGB Ⅰ was 30608 μg/g, which was significantly higher than that of CGB Ⅱ, while the coefficient of variation of CGB Ⅰ was also higher, indicating that Cd was strongly enriched in the soil of CGB Ⅰ with significantly spatial-temporal heterogeneity.

**Table 1:** Descriptive statistical analysis of Cd.

	Minimum	Median	Maximum	Mean	CV
CGB Ⅰ	66.75	295.45	30608.49	1131.55	323.44
CGB Ⅱ	30.89	236.24	1528.20	392.68	98.70
CGB Ⅰ-China	11	137	45984	147	/
CGB Ⅱ-China	14	125	33444	132	/
Continental crust [44]	/	80	/	/	/
Soil in Europe [45]	/	145	/	284	/
Floodplain sediments in Europe [45]	/	300	/	564	/

/ no data; CV: Coefficient of variation.

### 3.2 Spatial-temporal distribution of Cd

**Fig 2:**
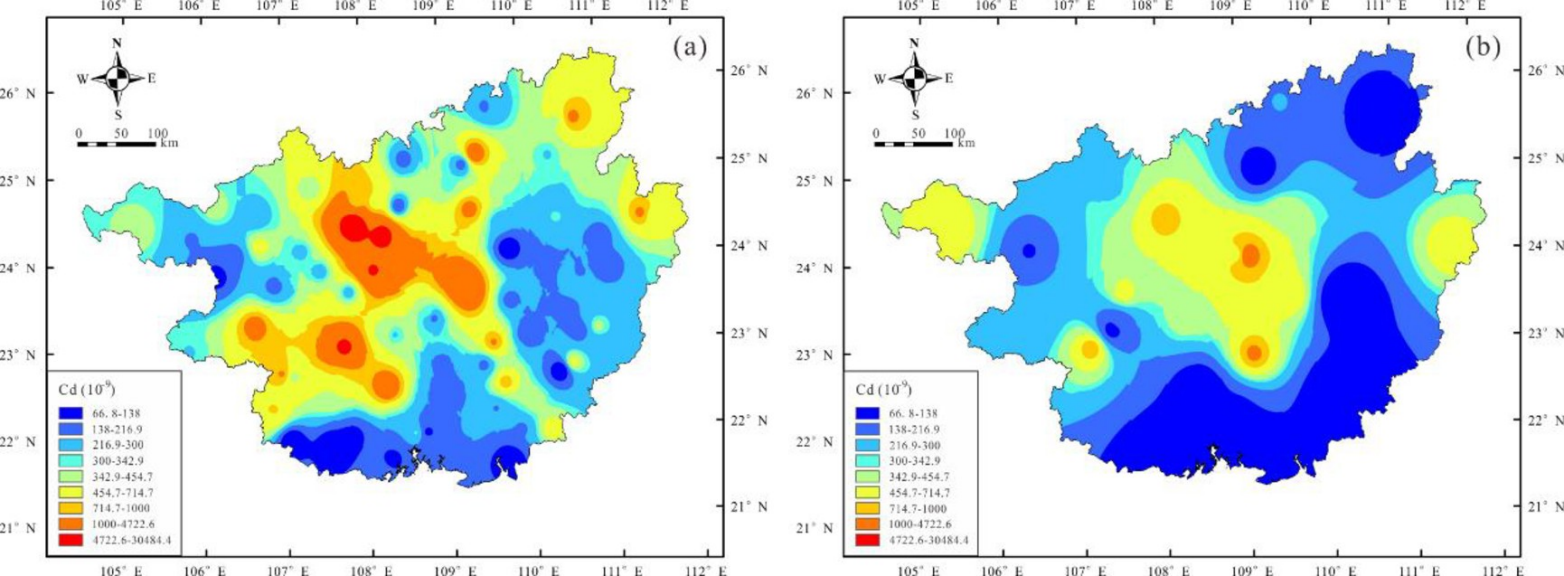
Guangxi Cd geochemical maps (a: CGB Ⅰ, b: CGB Ⅱ).

Cd content was interpolated using the inverse distance weighting method, and geochemical maps were drawn using ArcGIS 10.4 for CGB Ⅰ and CGB Ⅱ (Fig 2). The study area was divided into three parts based on Cd content according to soil contamination standards in China: strongly Cd-enriched areas (Cd content > 1000 μg/g), moderately Cd-enriched areas (Cd content between 300 μg/g and 1000 μg/g), and Cd background areas (Cd content < 300 μg/g). The Cd soil content from CGB Ⅰ was high in the northwest and relatively low in the southeast. Cd was strongly enriched in 17% of the samples, in Hechi, Laibin, southwestern Nanning, southern Baise, and small parts of Liuzhou and Hezhou. Approximately 31% of the samples were moderately enriched in Cd, while half the samples had background levels. In comparison, in Laibin and Nanning, the ratio of Cd exceeding thresholds in CGB II decreased significantly, and the strongly Cd-enriched samples decreased to approximately 7%. Approximately 33% of the samples were moderately Cd-enriched while 60% were background samples.

### 3.3 Fitting parameters of GTWR

Table 2 lists the fitting parameters of GTWR. R^2^ indicates the degree to which the dependent variable is explained by the independent variable, and ranges from 0 to 1; the larger the value, the better the fit. In this research, the R^2^ was 0.85, indicating that the independent variable had good explanatory power for the dependent variable. The residual sum of squares in this study was 16.45. The Akaike information criterion (AICc) is one of the common indicators used to test the regression model and the goodness of fit; the smaller the value, the better the fit [46]. The spatial-temporal distance ratio was automatically set to 0.27 to minimize the AICc.

**Table 2:** Fitting parameters of GTWR.

AICc	R^2^	RSS	STDR
283.59	0.85	16.45	0.27

AICc: Akaike information criterion; RSS: Residual sum of squares; STDR: Spatial-temporal distance ratio.

### 3.4 Statistics of coefficients of driving factors

**Fig 3:**
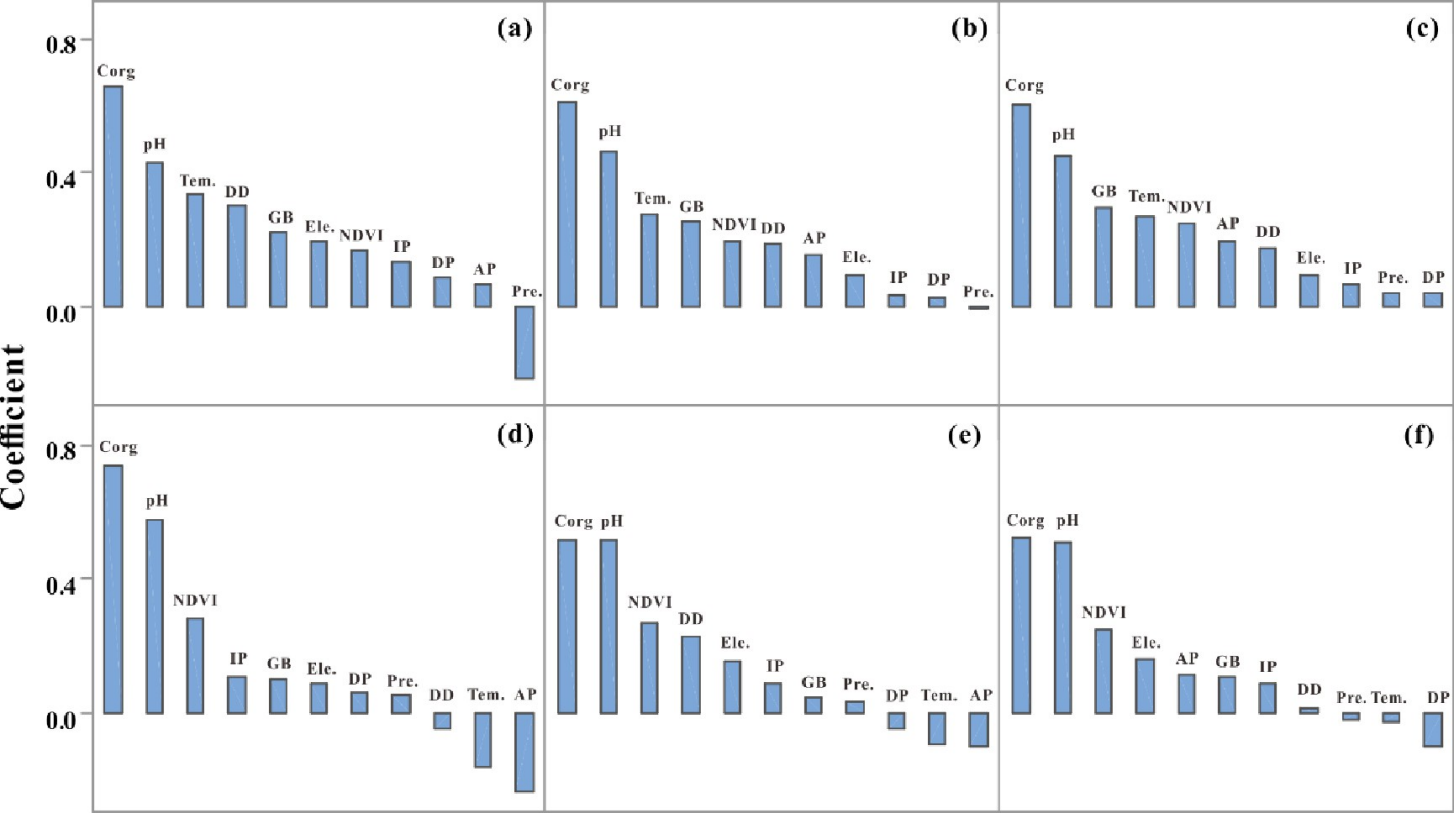
Coefficients of driving factors (Tem.: Temperature; Ele.: Elevation; GB: Geological background; DD: Deposit density; IP: Industrial pollutants; DP: Domestic pollutants; AP: Agricultural pollutants; (a) Strongly Cd-enriched area for CGB I; (b) Moderately Cd-enriched area for CGB I; (c) Cd background area for CGB I; (d) Strongly Cd-enriched area for CGB II; (e) Moderately Cd-enriched area for CGB II; (f) Cd background area for CGB II).

Regression coefficients indicate the degree of influence of each driving factor on the spatial-temporal variability of Cd. The positive and negative signs of the coefficients indicate positive and negative correlations between the variables and spatial locations, respectively, and the magnitude indicates the strength of the correlation. Fig 3 shows the median regression coefficients (coefficients of NDVI and Zr were analyzed and shown in absolute values) for different Cd content areas in CGB I and CGB II. The median coefficients of organic carbon and pH in the strongly Cd-enriched, moderately Cd-enriched, and background areas of CGB I were similar. Factors with high median coefficients in strongly Cd-enriched areas for CGB I were temperature, deposit density, geological background, and elevation. In addition, the top four factors responsible for the spatial variability of Cd in moderately Cd-enriched areas were, in descending order, temperature, geological background, NDVI, and deposit density. Factors with high median coefficients in the Cd background area include geological background, temperature, NDVI, and agricultural pollutants.

The key influencing factors for the spatial variation of Cd in strongly Cd-enriched area for CGB II were NDVI, industrial pollutants, geological background and elevation. The top four influencing factors in the moderately Cd-enriched area were NDVI, deposit density, elevation and industrial pollutants. The top four influencing factors in the background area were NDVI, elevation, agricultural pollutants, and geological background.

## 4 Discussion

### 4.1 Spatial-temporal variability of coefficients of natural variables

**Fig 4:**
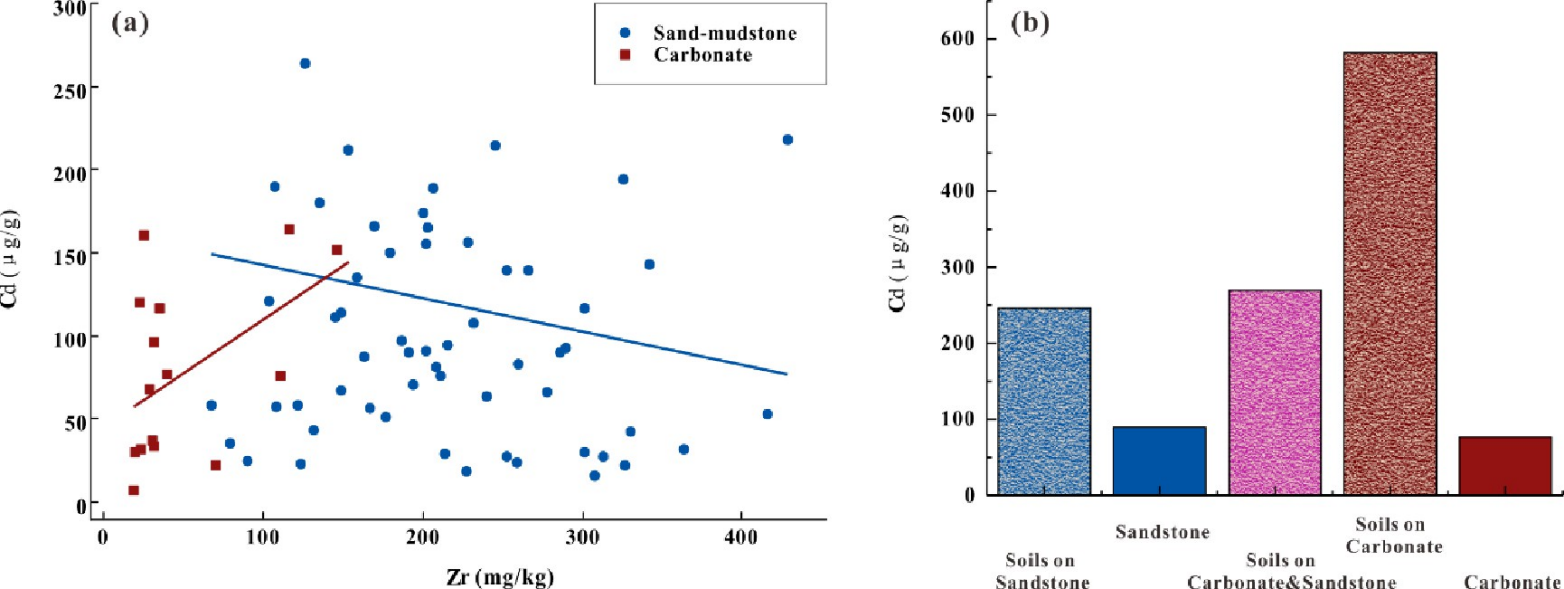
a: Concentrations of Cd and Zr in different rocks and soils; b: median Cd.

**Fig 5:**
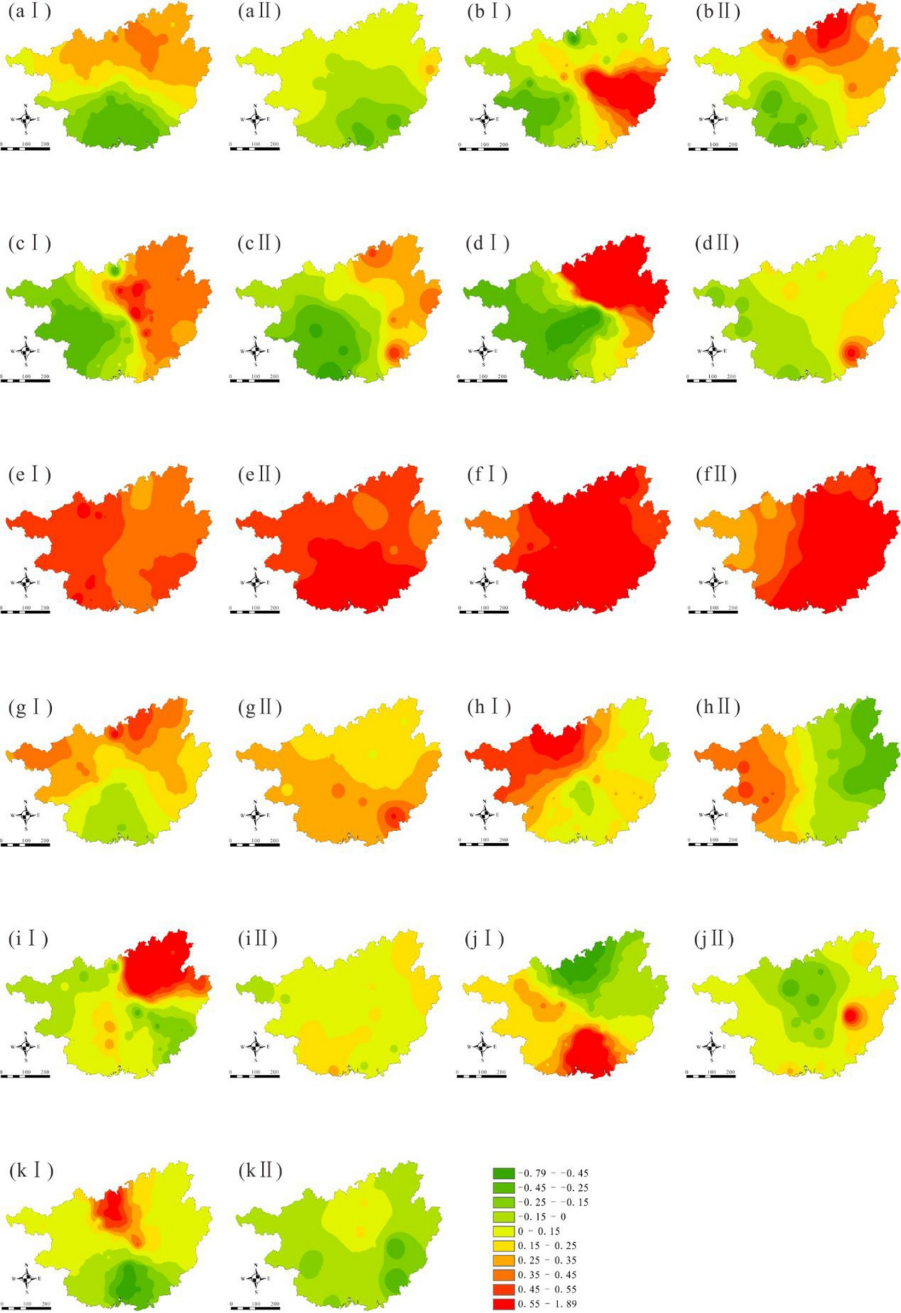
Spatial variability of regression coefficients in GTWR model (a: Geological background; b: Elevation; c: Temperature; d: Precipitation; e: pH; f: Corg; g: NDVI; h: deposit density; i: Industrial pollutants; j: Agricultural pollutants; k: Domestic pollutants; Ⅰ for CGB Ⅰ; Ⅱ for CGB Ⅱ).

Carbonate weathering is one of the main causes of Cd enrichment in karst areas. Our study showed that geological background played an important role in the spatial variability of Cd in both Cd-enriched and background areas (Fig 5). The results showed a significant positive correlation between geological background and Cd in soils developed on carbonate, a negative correlation between geological background and Cd in soils developed on sand-mudstones and intermediate-acidic rocks, and a poor correlation for soils developed on carbonate and sand-mudstone (Fig 4). Parent materials and the chemical weathering of rocks and minerals are important sources of Cd in soils [47–51]. The relationship between Zr and Cd was analyzed in 73 rocks collected from the study area (shown in Fig 4A). Significant positive correlations were identified between Zr and Cd contents in carbonate and negative correlations between Zr and Cd in sand-mudstones, indicating that the relationships between Zr and Cd in soils were similar to those in rocks. Fig 4B shows the median Cd content in sand-mudstone and carbonate, and the soils developed on sand-mudstone, carbonate, and carbonate-sand-mudstone in this study area. Although the Cd content in carbonate was very low (median 76 μg/g), Cd was highly enriched in soils (median 582 μg/g) developed on carbonate. The weathering rate of carbonate is much higher than that for the other rocks; the elements in carbonate are released more rapidly and thus more likely to cause secondary Cd enrichment. Moreover, major elements (such as Ca, Mg, K, and Na) were leached out. The carbonate content was greatly reduced by the decomposition and transformation of minerals in the bedrock during weathering [52,53], while the residual heavy metals were relatively enriched [54]. In addition, substantial clay materials and iron-manganese oxides formed during carbonate weathering, which have a strong adsorption capacity for heavy metals [55].

Temperature, precipitation, and elevation were positively correlated with Cd in the north and east of the study area and negatively correlated in the west and southwest. Climate affects the hydrothermal conditions of the soil, which in turn affects the direction and intensity of a range of physical, chemical, and biological processes in the soil [56–58]. [59] suggested that temperature and precipitation play integral roles in the soil formation process. Areas with good hydrothermal conditions (high temperature and precipitation) are dominated by chemical weathering [60], and more clay minerals, including kaolinite and iron-manganese oxides formed in the weathering products, which adsorb large amounts of heavy metals. Experimental studies have shown that heavy metals adsorbed on the soil surface migrate to their interior rapidly with increasing temperature, thus releasing the adsorption sites, and the adsorption capacity of soils for heavy metal ions is gradually enhanced. The topography also plays an important role in soil formation. [61] developed a landscape evolution model to study the soil formation process and considered the transport of materials during soil formation as a function of elevation evolution over time. In hilly uplands, where slopes are steeper and surface runoff is greater, there is greater weathering, transport, and denudation. Thus, elevation is positively correlated with Cd [62].

As discussed above, soil-forming parent materials and soil-forming factors can lead to spatial variability of heavy metals. However, long-term soil-forming processes may not influence the short-term temporal variability of Cd. In the study area, precipitation increased significantly in CGB II, and its influence coefficient increased correspondingly (Fig 5), suggesting that precipitation may influence Cd variability in various ways. Studies have shown that heavy metals may enter the atmosphere through fossil fuel combustion and industrial smoke, and are adsorbed in aerosols, which then enter the soil through atmospheric wet deposition [63]. In recent years, atmospheric wet deposition has been recognized as one of the main sources of heavy metals in soils on a large scale [64–66]. The results showed that the coefficient of precipitation in the eastern part of the study area was positive, and northeastern Guangxi was adjacent to Hunan Province, which is also rich in non-ferrous minerals. Therefore, Cd in nearby soils may originate from wet deposition. Precipitation also affects the migration and transport of heavy metals in the soil [67]. As the intensity and frequency of precipitation increases, the leaching of heavy metals from the soil increases, and these leached heavy metals enter the river system or infiltrate into groundwater [68]. Therefore, the negative correlation between precipitation and spatial variation of Cd may be related to the leaching effect. In addition, precipitation gradually increases with increasing elevation but decreases after reaching a maximum at a certain elevation. This may also explain the positive correlation between elevation and Cd concentrations. Moreover, anthropogenic activities are intensive at lower altitudes and introduce high concentrations of heavy metals, which is one of the reasons for the negative coefficient of elevation.

Both pH and organic carbon were positively correlated with Cd over the entire study area. The pH, an important indicator of the soil environment, has been widely reported to correlate with heavy metals [69]. Studies have shown that the negative charges on the surface of clay minerals, hydrated oxides, and organic matter in soil increase with pH, and thus the adsorption of heavy metal ions is enhanced. However, when the soil pH is too high, heavy metal precipitates are leached out by complexation with hydroxyl groups, resulting in a decrease in heavy metal content in the soil [70]. Organic carbon is the main occurrence medium for Cd and plays an important role in the mobility and effectiveness of Cd in soils. Under most conditions, organic acids are present in the form of negative ions that react strongly with Cd ions to immobilize Cd in the soil.

The NDVI of CGB Ⅱ was significantly higher than that of CGB Ⅰ. In addition, the results showed that NDVI was negatively correlated with Cd for CGB Ⅰ, and the NDVI coefficients were negative for CGB II (Fig 5), with larger absolute values compared to CGB Ⅰ, which is consistent with previous results [71]. The increased NDVI suggested improved vegetation cover, which can maintain soil and water by regulating surface runoff, reducing desertification and soil erosion in the watershed, and reducing pollutants transported from upstream. Although NDVI, which reflects vegetation cover, is regarded as a natural factor, its improvement depended on changes in the natural environment, such as precipitation, temperature, and illumination, as well as the level of environmental protection, afforestation, and the implementation of sustainable development strategies.

### 4.2 Spatial-temporal variability of coefficients of anthropogenic variables

Deposit density was mostly positively correlated with Cd for CGB Ⅰ, mainly in Hechi and its surroundings, where mining and smelting have resulted in serious Cd contamination of soils. The coefficients of deposit density were the largest in strongly Cd-enriched areas and relatively small in moderately Cd-enriched and background areas. In comparison, the degree and extent of the impact of mining on CGB Ⅱ decreased significantly. Cd is a typical dispersed element that mainly exists in isomorphic sphalerite and wurtzite in Pb-Zn deposits. There are numerous Pb-Zn deposits in Hechi and a large amount of waste ore, tailings, and silt deposited along the banks and at the bottom of the riverbed of the Dahuanjiang River owing to the piling of tailings and slag, which causes serious contamination of the surrounding soil. In 2001, a rainstorm washed out the tailings ponds of more than 30 plants upstream, flooding the farmland downstream of the Dahuanjiang River and contaminating the coastal soil with heavy metals [72,73]. In contrast, the impact of mining decreased for CGB Ⅱ. Although mining activities were nearly unchanged from 2010 to 2016, the pollutants emitted by mining and refining may have been reduced with the greater emphasis on environmental protection.

The discharge of industrial wastewater in Guangxi Province was approximately 1 billion tons in 2010, and the spatial distribution of soil Cd was affected by industrial wastewater for CGB Ⅰ, especially at Liuzhou in the northeast of the study area. In contrast, the discharge of industrial wastewater decreased significantly to approximately 360 million tons in 2016, and the coefficients of industrial wastewater in the strongly Cd-enriched area were significantly reduced. However, the coefficients of industrial wastewater in the moderately Cd-enriched and background areas were slightly increased for CGB Ⅱ. Liuzhou is the industrial center of Guangxi, comprising more than 120 coal consuming enterprises with an annual coal consumption of 12 million tons. Moreover, in 2010, there were iron and steel, metallurgy, chemical industry, power generation and other enterprises, as well as many zinc metallurgy enterprises in Liuzhou [74]. The discharge of industrial wastewater in Liuzhou City was 230 million tons in 2010, which decreased to 140 million tons in 2016. The temporal variability of Cd correlated with changes in industrial wastewater discharge.

The total amount of agricultural pollutant emissions (the sum of chemical oxygen demand emissions and ammonia nitrogen emissions) was 250000 tons in 2010 and approximately 320000 tons in 2016. The area with the strongest agricultural influence was located in the south of the study area for CGB I. By contrast, the range of influence of agricultural pollution for CGB II was reduced. The elevation of Guangxi is high in the north and low in the south, and the proportion of agricultural land in the south is higher. Many studies have shown that the contribution of agricultural sources to soil Cd mainly originates from phosphorus fertilizer. Schroeder and Balassa first reported the relationship between phosphate fertilizer application and Cd in 1963. Later, Australia [75], the United States [76], Norway [77], Denmark [78] (Christensen, 1991), Finland [79], Britain [80], China [81,82] and other countries also reported Cd accumulation potentially caused by phosphorus fertilizer application. Phosphate fertilizer is mainly produced by the chemical treatment of phosphate rock, which contains varying degrees of Cd. Globally, the total average Cd content of sedimentary phosphate rock and igneous phosphate rock is 21 mg/kg (<1–150 mg/kg) and 2 mg/kg (<1–5 mg/kg), respectively, and the former accounts for approximately 85% of the total output of phosphate fertilizer [83]. The average content of Cd in 36 phosphate rocks in China is 15.3 ± 74 mg/kg (0.1–571 mg/kg), and the average content of Cd in 30 phosphate rock production plants is 0.60 ± 0.63 mg/kg (0.1–2.93 mg/kg) [84]. Recent studies report that the Cd content of phosphorus fertilizer and NPK fertilizer produced in China is approximately 1.5–3.2 mg/kg and 0.5–1.5 mg/kg [85,86], respectively. The content of Cd in phosphate fertilizers produced in China is much lower than that produced in North America (16–45 mg/kg) [87]. Our research showed that the influence coefficient of agricultural pollution gradually increased from the strongly Cd-enriched area to the moderately Cd-enriched area and then to the background area, and that agricultural pollution was the most important factor influencing the variability of Cd relative to other anthropogenic factors in the background area. Based on the above discussions, we can conclude that the application of phosphorus fertilizer could lead to the accumulation of Cd, but the contribution was minor relative to mining and industrial pollutants.

The problem of excessive heavy metals in domestic sewage is a concern [88,89]. However, our study showed that the impact of domestic sewage was weaker than that of the other factors. Moreover, the total domestic sewage discharge for CGB I was 1.2 billion tons, while that for CGB II was approximately 1.6 billion tons, but the influence range and intensity for CGB II were weakened relative to CGB I. Although the discharge of domestic sewage increased, its impact intensity decreased, which may be due to the improved sewage treatment capacity and the reduced Cd content in sewage for CGB II. In addition, certain anthropogenic variables were negatively correlated with Cd, as the industrial emissions or agricultural pollutants in certain region had a lower Cd content.

### 4.3 Driving factors of spatial-temporal variability of Cd

In summary, pH and organic carbon were the main factors influencing Cd and were strongly spatially correlated with Cd. Soil-forming factors, dominated by temperature, were the most important factors controlling the spatial variability of Cd, as these factors influence the strength and rates of rock weathering. Soil-forming parent materials provide the initial source of Cd and are strongly spatially correlated with Cd, especially in moderately Cd-enriched and Cd-background areas. The significant enrichment of Cd in CGB I was mainly due to mining and refining activities. In CGB II, industrial pollutants became the major anthropogenic factor controlling the Cd content in significantly Cd-enriched areas. NDVI, which indicates improved vegetation cover and reduced desertification, was clearly linked to reduced Cd content owing to the reduction of soil erosion in the watershed and the consequent reduction of pollutants transported from upstream. The spatial-temporal variability of Cd is a comprehensive result of these driving factors.

## Conclusions

Cd in the study area generally showed higher distribution in the northwest and lower distribution in the southeast. Approximately 17% of the samples were significantly enriched in Cd, mainly distributed in Hechi, Laibin, southwest of Nanning, south of Baise, and a small part of Liuzhou and Hezhou; 31% of the samples were moderately enriched in Cd, and approximately half of the samples were located in the Cd background area for CGB I. In contrast, the ratio of Cd exceeding the thresholds decreased significantly for CGB II with 7% significantly enriched Cd. Approximately 33% of the samples were moderately Cd-enriched, and approximately 60% had background Cd levels.

The spatial-temporal variability of Cd was the result of the coupling effect of multiple factors. Spatially, pH and organic carbon, as the main reservoirs for and influencing factors of Cd, were significantly positively correlated with Cd. Soil-forming factors that were dominated by temperature were strongly correlated with Cd levels. Because of the special geochemical properties of carbonates, their weathering products were significantly enriched in Cd; thus, the geological background played a significant role in Cd variability, especially in the Cd background area. Anthropogenic activities, including mining and industrial emissions, as point sources, were spatially correlated with Cd in significantly Cd-enriched areas, whereas agricultural pollutants and domestic pollutants were minor sources of Cd pollution in this study area.

Anthropogenic activity was the main factor influencing the temporal variation of Cd at the regional scale, with mining causing significant Cd enrichment in CGB I, and industrial emissions being the primary factor for Cd enrichment in CGB II. NDVI indicates changes in the natural environment and is attributed to increased levels of environmental protection. NDVI is significantly negatively correlated with the temporal variability of Cd due to the maintenance of heavy metals by vegetation.

Study of the distribution characteristics of heavy metals and their changing trends on large spatial-temporal scales and quantifying their driving factors is important for understanding the geochemical behavior of heavy metals to inform the prevention and control of heavy metal pollution. According to this study, mining activities and factory emissions are important sources of Cd, thus it is necessary to take multiple management measures to reduce and remove the pollutants in the waste. Moreover, improving vegetation coverage is one of the other effective measure to conserve water and soil and reduce Cd content. There is a need for long-term monitoring of the spatial-temporal variability of heavy metals to clarify the impact of anthropogenic disturbance on the natural environment.
